# Genome-wide association analysis of cystatin-C kidney function in continental Africa

**DOI:** 10.1016/j.ebiom.2023.104775

**Published:** 2023-08-26

**Authors:** Richard Mayanja, Tafadzwa Machipisa, Opeyemi Soremekun, Abram B. Kamiza, Christopher Kintu, Allan Kalungi, Robert Kalyesubula, Obondo J. Sande, Daudi Jjingo, June Fabian, Cassianne Robinson-Cohen, Nora Franceschini, Dorothea Nitsch, Moffat Nyirenda, Eleftheria Zeggini, Andrew P. Morris, Tinashe Chikowore, Segun Fatumo

**Affiliations:** a The African Computational Genomics (TACG) Research Group, MRC/UVRI and LSHTM Uganda Research Unit, Entebbe, Uganda; b Department of Immunology and Molecular Biology, School of Biomedical Sciences, Makerere University, College of Health Sciences, Kampala, Uganda; c Department of Medicine, University of Cape Town & Groote Schuur Hospital, Cape Town, South Africa; d Clinical Research Laboratory-Genetic and Molecular Epidemiology Laboratory (CRLB-GMEL), Population Health Research Institute (PHRI) & McMaster University, David Braley Cardiac, Vascular and Stroke Research Institute, 237 Barton Street East, Hamilton, Ontario, L8L 2X2, Canada; e Malawi Epidemiology and Intervention Research Unit, Lilongwe, Malawi; f Medical Research Council/ Uganda Virus Research Institute (MRC/UVRI) and London School of Hygiene and Tropical Medicine (LSHTM) Uganda Research Unit, Entebbe, Uganda; g African Center of Excellence in Bioinformatics (ACE-B), Makerere University, Kampala, Uganda; h Medical Research Council/Wits University Rural Public Health and Health Transitions Research Unit (Agincourt), School of Public Health, Faculty of Health Sciences, University of the Witwatersrand, Johannesburg, South Africa; i Wits Donald Gordon Medical Centre, School of Clinical Medicine, Faculty of Health Sciences, University of the Witwatersrand, Johannesburg, South Africa; j Division of Nephrology and Hypertension, Department of Medicine, Vanderbilt University Medical Center, Nashville, TN, USA; k Gillings School of Global Public Health, University of North Carolina, Chapel Hill, NC, USA; l London School of Hygiene and Tropical Medicine London, UK; m Institute of Translational Genomics, Helmholtz Zentrum München - German Research Center for Environmental Health, Neuherberg, Germany; n TUM School of Medicine, Translational Genomics, Technical University of Munich and Klinikum Rechts der Isar, Munich, Germany; o Centre for Genetics and Genomics Versus Arthritis, Centre for Musculoskeletal Research, Division of Musculoskeletal and Dermatological Sciences, The University of Manchester, Manchester, UK; p Sydney Brenner Institute for Molecular Bioscience, Faculty of Health Sciences, University of the Witwatersrand, Johannesburg, South Africa; q MRC/Wits Developmental Pathways for Health Research Unit, Department of Pediatrics, Faculty of Health Sciences, University of the Witwatersrand, Johannesburg, South Africa

**Keywords:** Cystatin-C, Estimated glomerular filtration rate, Kidney function, Genome-wide association study, Fine-mapping, Continental Africa

## Abstract

**Background:**

Chronic kidney disease is becoming more prevalent in Africa, and its genetic determinants are poorly understood. Creatinine-based estimated glomerular filtration rate (eGFR) is commonly used to estimate kidney function, modelling the excretion of the endogenous biomarker (creatinine). However, eGFR based on creatinine has been shown to inadequately detect individuals with low kidney function in Sub-Saharan Africa, with eGFR based on cystatin-C (eGFRcys) exhibiting significantly superior performance. Therefore, we opted to conduct a GWAS for eGFRcys.

**Methods:**

Using the Uganda Genomic Resource, we performed a genome-wide association study (GWAS) of eGFRcys in 5877 Ugandans and evaluated replication in independent studies. Subsequently, putative causal variants were screened through Bayesian fine-mapping. Functional annotation of the GWAS loci was performed using Functional Mapping and Annotation (FUMA).

**Findings:**

Three independent lead single nucleotide polymorphisms (SNPs) (P-value <5 × 10^−8^ (based on likelihood ratio test (LRT))) were identified; rs59288815 (*ANK3),* rs4277141 (*OR51B5*) and rs911119 (*CST3*). From fine-mapping, rs59288815 and rs911119 each had a posterior probability of causality of >99%. The rs911119 SNP maps to the cystatin C gene and has been previously associated with eGFRcys among Europeans. With gene-set enrichment analyses of the olfactory receptor family 51 overlapping genes, we identified an association with the G-alpha-S signalling events.

**Interpretation:**

Our study found two previously unreported associated SNPs for eGFRcys in continental Africans (rs59288815 and rs4277141) and validated a previously well-established SNP (rs911119) for eGFRcys. The identified gene-set enrichment for the G-protein signalling pathways relates to the capacity of the kidney to readily adapt to an ever-changing environment. Additional GWASs are required to represent the diverse regions in Africa.

**Funding:**

10.13039/100004440Wellcome (220740/Z/20/Z).

**Research in context**

**Evidence before this study**

Reported CKD prevalence varies greatly within Africa, in part, due to the heterogeneity of study designs and different criteria used for diagnosing CKD. Creatinine-based estimated glomerular filtration rate (eGFRcrea) are the most widely used tests for assessing kidney function and considerably cheaper than cystatin-C-based estimated glomerular filtration rate (eGFRcys) despite evidence that eGFRcys is a better biomarker in African populations. While CKD has both infectious and noncommunicable causes in Africa, little is known about the impacts of genetic, environmental, and toxin exposures. For example, Zhang et al., 2021 estimated the heritability of chronic kidney CKD to fall within a range of 25%–44% with higher estimates in individuals of African ancestry. To date, there is only one genome-wide association study (GWAS) of kidney function in continental African populations which used serum creatinine as the biomarker for eGFR. There were some key limitations. First, the sample size was small (N = 3288). Second, the biomarker used (serum creatinine) is prone to influences from variations in muscle mass, levels of animal protein ingestion, sex, age, and tubular secretion. In this study we carried out a GWAS of kidney function using eGFRcys (a more robust and sensitive kidney biomarker) in 5877 continental Africans.

**Added value of this study**

We identified two genome-wide significant lead single nucleotide polymorphisms (SNPs) (rs59288815 in the *ANK3* and rs4277141 in the *OR51B5* intronic regions) that have not been previously associated with eGFRcys in other populations to the best of our knowledge. Furthermore, our study also replicated the rs911119 SNP in the *CST3* gene that was previously associated with eGFRcys among individuals of European ancestry.

**Implications of all the available evidence**

Our work shows an association between eGFRcys and three loci (rs59288815 (*ANK3*), rs4277141 (*OR51B5*) and rs911119 (*CST3*)) in Ugandans. By incorporating biomarkers such as serum cystatin-C, we can unlock previously unknown information about kidney function and potential disease mechanisms. Using all the available evidence, we have uncovered genetic associations with cystatin-C among different populations, revealing the importance of exploring diverse populations in studying genetic diseases. These discoveries open the door to new prevention and personalized medicine strategies, ultimately transforming how we approach kidney function biomarkers in genomic studies.

## Introduction

In Africa, the estimated prevalence of chronic kidney disease (CKD) varies from 13 to 15%, compared with the global average of 10%. It is probable that some genetic factors associated with kidney function are specific to African populations and others are shared. Since the prevalence and severity of kidney disease varies across different populations, it might not be reliable to generalize findings from genetic studies conducted in Europeans to other populations including Africans^1^

To evaluate kidney function, biomarkers such as serum creatinine (Scr), cystatin-C (sCys-C), albuminuria, and blood urea nitrogen (BUN) can be used.^2^ In clinical practice, only Scr-based estimated glomerular filtration rate (eGFRcrea) is commonly used as a biomarker for kidney function because it is cost-effective and widely available. Hence, the only known genome-wide association study (GWAS) conducted on kidney function in Africa, used Scr as a biomarker to identify genetic factors associated with kidney function in a sample of 3288 individuals from Uganda.^3^ However, recent literature has shown that eGFRcrea estimation is limited in Sub-Saharan Africa, and in particular, that eGFRcrea misses people with reduced kidney function whilst sCys-C-based estimated glomerular filtration rate (eGFRcys) correlated with the gold-standard iohexol kidney function measurement.^4^ We know from high-income settings that there are clinical scenarios in which eGFRcrea may not accurately represent kidney function, such as in individuals with low muscle mass or obesity.^5^ However, these did not explain the discrepancy between eGFRcrea and the gold standard kidney function test. In high income settings, sCys-C is also more sensitive than Scr in detecting the early stages of kidney disease. However, eGFRcys proved in Sub-Saharan Africa to not only be better at detecting early stages of kidney disease, but also to detect more advanced CKD when compared to eGFRcrea.^4^ In high income settings, cystatin C has been shown to be influenced by adiposity, white blood cell count, underlying inflammation, thyroid disease, as well as glucocorticoid treatment.^6^ Of these, inflammation and increased white cell count may be more prevalent in areas with endemic infections.^7^ But, sCys-C levels are less influenced by ethnicity than Scr levels, which makes it a more accurate biomarker in individuals from diverse genetic backgrounds.^8^ Thus, there is a need to add a more sensitive and robust biomarker in Sub-Saharan Africa, the sCys-C-based estimated glomerular filtration rate (eGFRcys),^9^ as well as a genetic study on eGFRcys in Africans. In this study, we set out to identify genetic variants that are associated with kidney function using estimated glomerular filtration rate (eGFR) based on sCys-C levels in samples from participants of the Uganda Genome Resource (UGR), which is part of the Uganda General Population Cohort (GPC).^10^^,^^11^

## Methods

### The Uganda genome resource study

The individuals included in this study were selected from the UGR which is part of GPC, that has been described by previous studies.^11^ Briefly, GPC is a population-based cohort comprising around 22,000 residents from 25 neighbouring villages in Kyamulibwa sub-county, part of Kalungu district in rural Southwest Uganda. The GPC was established in 1989 to primarily investigate the incidence and prevalence of HIV infection in Uganda. It has since expanded its scope to investigate the genetics and epidemiology of various communicable and non-communicable diseases. During a survey conducted in the research study area, samples were collected from the research participants. The study area is clustered into villages defined by governmental borders and ranges in size from 300 to 1500 inhabitants and includes numerous households. In 2011, the University of Cambridge, Wellcome Sanger Institute (WSI), and MRC/UVRI in Uganda collaborated on the GPC Round 22 study to provide aetiological insights into the genetic variation of communicable and non-communicable diseases. It was contained within one annual survey round of the longitudinal cohort. It involved five primary stages in 2011: mobilization (recruitment and consent), mapping, census, survey, and feedback of results and clinical follow-up. The census consisted of two questionnaires, one for families and one for individuals, which collected information on socio demographics data and household members, respectively. A standard questionnaire was used to collect lifestyle and health information, including biophysical measurements and blood samples. In this study, 5000 samples were genotyped, and 2000 samples were sequenced from nine ethnolinguistic groups from the GPC. Therefore, the genetic data from GPC is referred to as the UGR.^3^^,^^12^

### Ethics statement

The Uganda GPC was approved by the Science and Ethics Committee of the Uganda Virus Research Institute Research (UVRI-REC -#HS 1978) and the Uganda National Council for Science and Technology (UNCST -#SS 4283).

### Genotyping and quality control

Genotyping and quality control were performed in previous studies.^3^^,^^12^ In brief, 5000 individuals were genotyped on the Illumina HumanOmni2.5-8 array and 4772 remained after quality control.

**Fig. 1:**
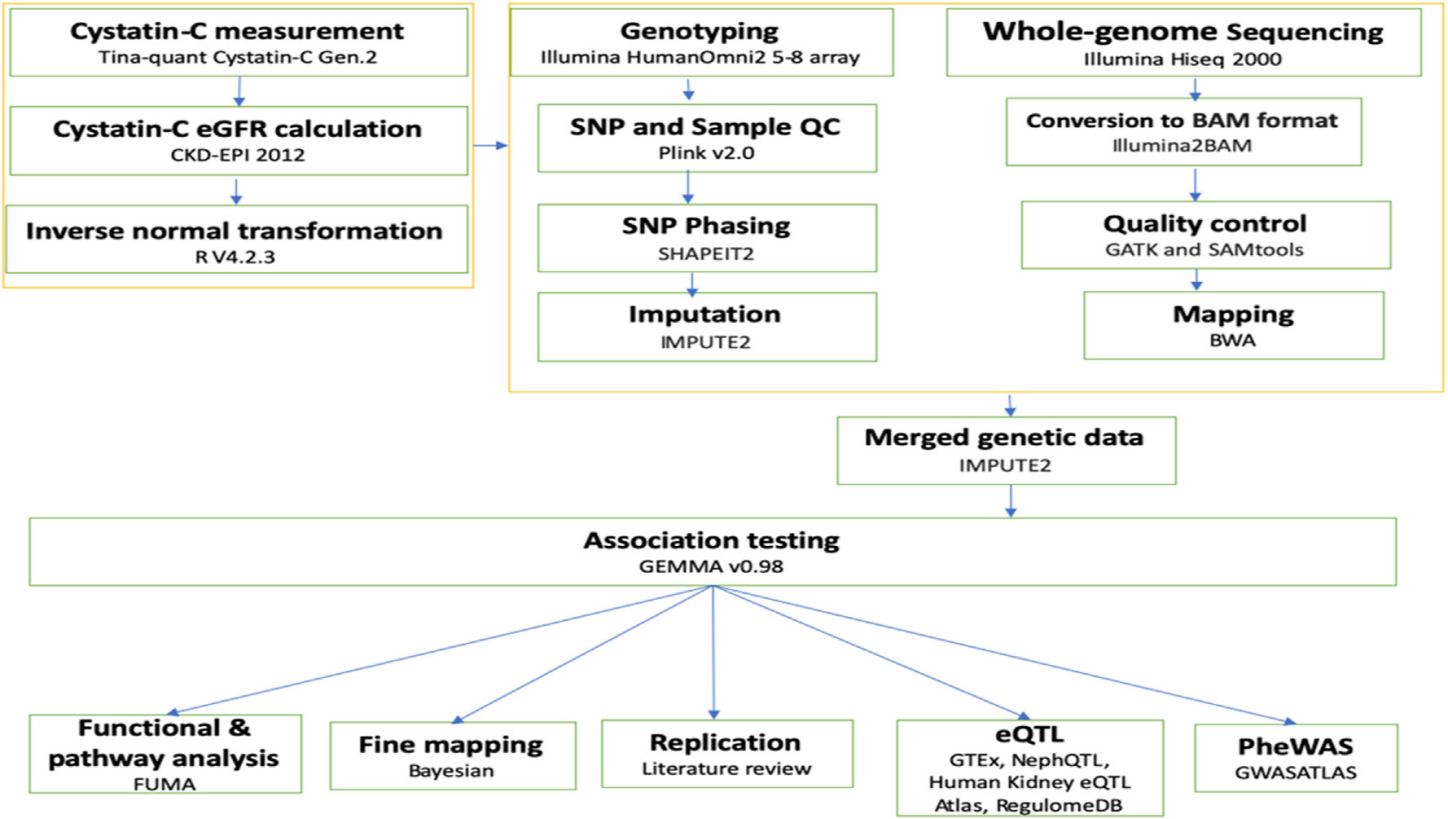
Detailed flowchart of the study analyses.

The genotyped data underwent imputation using the 1000 Genomes Project Phase 3 reference panel, which contains genotypes for approximately 37 million SNPs. Imputation was performed using the Michigan Imputation Server and the Minimac3 software. 2000 Ugandan samples underwent low-coverage whole-genome sequencing on the Illumina HiSeq 2000 using 75 bp paired-end reads, at low coverage of 4× for each sample. After quality control, 1978 sequenced samples remained; including the 343 individual samples that had been genotyped and sequenced. Genotyped data was excluded for 343 samples that were both genotyped and sequenced. The quality-controlled imputed genotype and the sequence data were then merged to create a single pooled dataset for analysis. In the merged dataset, we looked for any systematic differences between genotype and sequence calls. After doing quality control on the merged genotyping and sequence data, we performed principal component analysis to examine if any main components represented systematically different aspects of the chip and sequence data. For this analysis, the genotyping and sequence data were merged, and only overlapping variants were used. Principal components (PC) were then estimated on unrelated individuals (IBD< 0.10) for variants with frequencies greater than 5%, and those results were projected onto the remaining cohort. When we looked at the first 10 PCs, we found no evidence suggesting systematic differences between chip and sequence data. In addition, for the 343 individuals who had both sequence and genotype data, we plotted PCs principal components by projecting principal components from the genotype data onto the sequence data. The points were found to coincide, with no separation along PCs 1–10, indicating the absence of systematic differences between these data (Fig. 1).

### Laboratory testing of serum cystatin-C and estimated glomerular filtration rate calculation

The MRC/UVRI & LSHTM CDLS Immunochemistry laboratory conducted laboratory tests on bio-banked serum samples from GPC round 22 to measure sCys-C levels. The test used was the Tina-quant Cystatin-C Gen.2, which is a latex particle-enhanced immunoturbidimetric assay, and it was performed using the Roche Cobas 6000 Hitachi analyzer, Mannheim, Germany. The eGFRcys was determined using the Chronic Kidney Disease Epidemiology Collaboration.

(CKD-EPI) 2012 formula.^13^ After adjusting for age and sex, the eGFRcys residuals were transformed using the inverse rank normal method (Fig. 1).

### Association analysis

A GWAS was performed on 5877 samples with eGFRcys phenotypes out of the 6407 genotyped samples that previously passed quality control since some participants lacked phenotypic data (eGFRcrea). The univariant linear mixed model approach implemented by the Genome-wide Efficient Mixed Model Association algorithm (GEMMA) software version 0.96^14^ was applied. The input files for GEMMA included genotypes (BIMBAM), phenotypes (eGFR inverse transformed residues), SNP annotation and kinship matrix files. Subsequently, the 22 kinship matrices were generated earlier on in the UGR study^12^ using the leave-one-chromosome-out (LOCO) approach. To analyse SNPs along a chromosome for the association, LOCO generates a kinship matrix without including the chromosome being analysed. This was done sequentially for each chromosome to ensure that any causal SNPs on a specific chromosome are not included in the kinship matrix used to analyse that chromosome.^15^ Furthermore, we also applied a minor allele frequency (MAF) cut-off of 0.05 (imputation quality threshold) in the association analysis (Fig. 1).

### Replication of the lead single nucleotide polymorphisms

To determine if our top SNPs have previously been connected with Cystatin-C, we interrogated the GWAS catalog for SNPs that were already linked to cystatin-C. We also searched our lead SNPs in GWAS summary statistics from previously published studies such as Pattaro et al.*,* 2016,^16^ Stanzick et al., 2021^17^ and Sinnott-Armstrong et al., 2021.^18^

### Comparison of lead SNPs identified in UGR data using eGFRcys and eGFRcrea

To investigate whether using different biomarkers led to the identification of similar or distinct SNPs, we compared the SNPs previously linked to kidney function with eGFRcrea^3^ in the UGR dataset^11^ to those we identified with eGFRcys in the same dataset.

### Bayesian fine-mapping

Bayesian fine-mapping to identify potential causal variants for the locus ± 1 Mb of the lead SNPs was performed [15], and Z score was used to compute the Bayes factor for each SNP (BFi).

Below is the equation we used:

$$BFi=e^{\left[\frac{ziˆ2}{2}\right]}$$

We calculated the posterior probability of driving the association for each SNP using the equation below.

Posterior probability $=\frac{BFi}{\sum jBFj}$ The summation in the denominator is the total Bayes factor at SNPs at the locus.

The process of determining a credible set size with a 99% level of accuracy involved sorting the SNPs at the locus from highest Bayes' factor to lowest Bayes’ factor and counting the minimum number of SNPs required to reach a cumulative posterior probability of 0.99 or higher. We then constructed the 99% credible set of SNPs that account for 99% of the posterior probability of driving the association at each locus. Regional association plots were then generated for each of our lead SNP using the Locus zoom.^19^

### Colocalization of eGFR association signals with expression quantitative trait loci (eQTLs)

To determine if multiple genetic variants in the region of our lead SNPs are associated with kidney function, we performed colocalization analysis. We examined kidney eQTL data from various sources, including GTEx, NephQTL, the Human Kidney eQTL Atlas, and RegulomeDB. We compared the correlations between SNPs and tissue-specific gene expression levels within the regions of our lead SNPs. We evaluated genetic variation in densely genotyped human tissues within these regions. By analysing global RNA expressions within individual tissues, we treated gene expression levels as quantitative traits and identified eQTLs that showed a high correlation with genetic variation.^20^

### Functional mapping and annotation

We used gene-based tests and gene-set analysis to summarise SNP relationships at the gene level and associated them with biological pathways. Gene-based P-values were obtained using Multi-marker Analysis of GenoMic Annotation (MAGMA), which employs multiple linear regression. Gene set P-value was calculated using the gene-based P-value including canonical pathways and Gene Ontology (GO) terms from Molecular Signatures Database (MSigDB) v7.2.^21^ The default MAGMA v1.08 setting, which includes the SNP-wise model for gene analysis and competitive model for gene set analysis, was employed for both analyses, and the Bonferroni correction (gene) was used to account for multiple testing. We used 1000G phase 3 AFR as a reference panel to determine linkage disequilibrium (LD) across SNPs and genes. We utilised MAGMA gene-based analysis and gene-set analysis on the complete GWAS input data to conduct functional mapping and annotation of genetic correlations with Functional Mapping and Annotation (FUMA).^22^ Additionally, we examined genes that were prioritized by SNP2GENE and overrepresentation in various gene sets throughout the GENE2FUNC procedure. The main goal of GENE2FUNC is to provide information on the expression of prioritized genes and test for enrichment of the set of genes in pre-defined pathways. By integrating mapping approaches with biological pathway and enrichment tests, FUMA enables the prioritization of genes most likely to be involved in the trait of interest or gene sets associated with biological pathways involved in the development of traits.

### Phenome-wide association studies

A phenome-wide association study (PheWAS) was performed to investigate potential associations between a set of curated human phenotypes (phenome) and the lead eGFRcys variants. The goal was to gain insight into the relationship between our lead SNPs and various human phenotypes using data from independent sources obtained using GWASATLAS.^23^ PheWAS plots for our lead SNPs were generated by searching for their rsID numbers in GWASATLAS, which only considers SNPs with a P-value <0.05 for the analysis. SNPs on the same genomic coordinate are treated as identical despite differences in alleles across GWAS, and the chromosome and position of the rsID are obtained from dbSNP build 146 when the rsID is provided. Following the PheWAS analysis, the results of multiple hypothesis tests were reported as P-value = 0.05/number of tests.

### Gene expression analysis from previous studies

We searched the literature for studies that looked at gene expression in the kidney. Methods for analysing gene expression included studies that combined quantitative transcriptomics analysis (RNA-Seq) with antibody-based profiling of the same tissues to classify the tissue-specific gene expressions across major human organs and tissues (Fig. 1).

### Role of funders

Funders did not have any role in study design, data collection, data analyses, interpretation, or writing of report.

## Results

### The study participant characteristics

We performed our analysis on data from 5877 participants from the UGR^12^; 3373 females and 2504 males. The mean age was 35 years in females and 33 years in males. The average weight of females was 53 kg and for males was 52 kg. In terms of kidney function, we found that more females (141) than males (84) had eGFRcys levels less than 60 ml/min/1.72 m^2^ (Table 1).

**Table 1:** GPC participant characteristics.

Characteristics	Sex (N = 5877)	
Demographics	Males	Females
Age (years), mean (SD)	33 (19)	35 (18)
eGFRcys levels (min/1.72/min^2^), mean (SD)	120 (79)	116 (78)
Weight (kg), mean (SD)	52 (11)	53 (11)
Number of individuals with eGFRcys <60 (min/1.72/min^2^)	84	141
**Total**	**2504**	**3373**

### Findings from the genome-wide association study

**Fig. 2:**
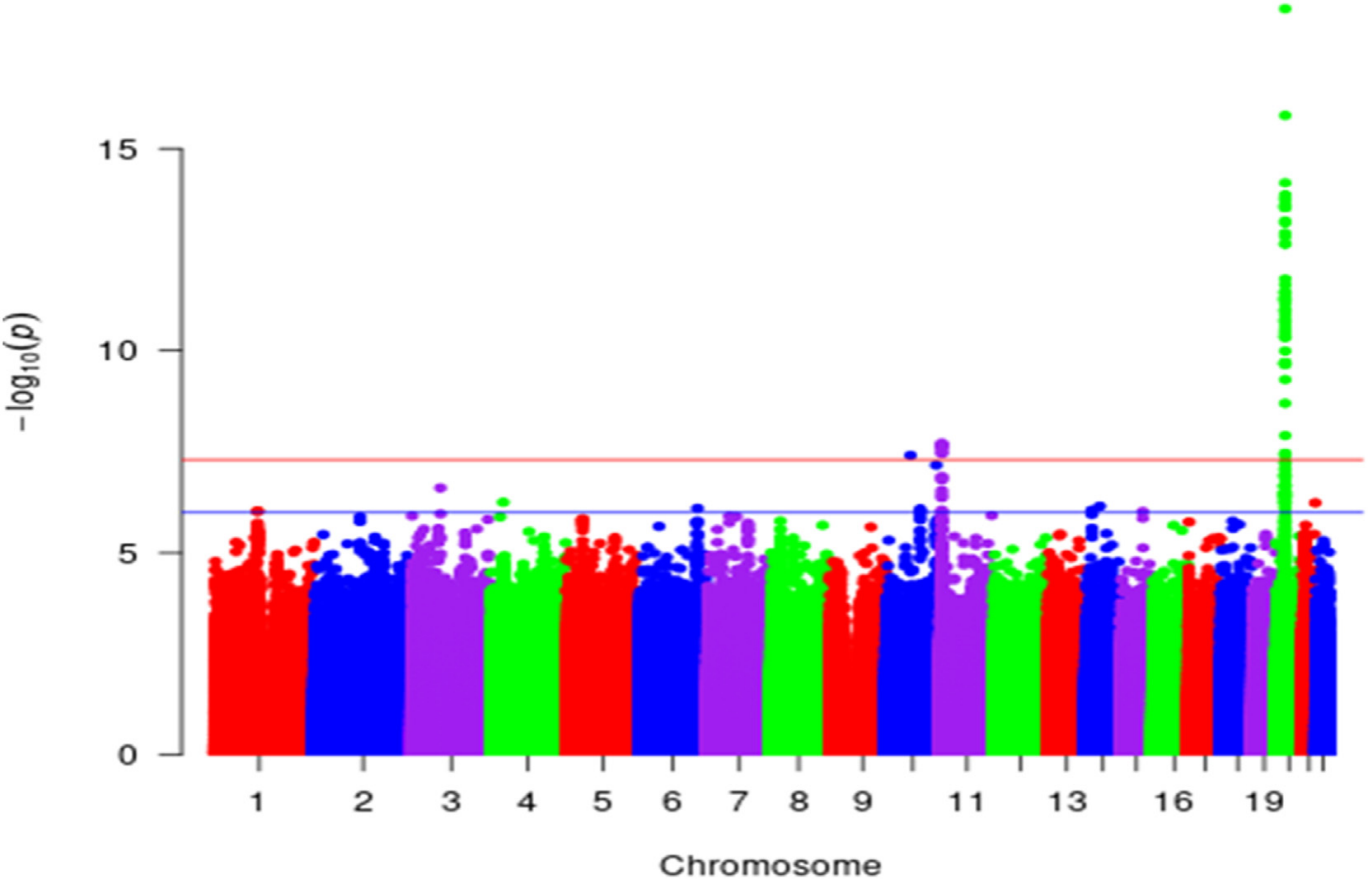
Manhattan plot for the UGR GWAS of eGFRcys (n = 5877). The red line indicates the genome-wide significance level threshold (P < 5 × 10^−8^ (based on LRT)) and the blue line indicates a suggestive genome-wide significance level of P < 5 × 10^−6^ (based on LRT).

**Fig. S1:**
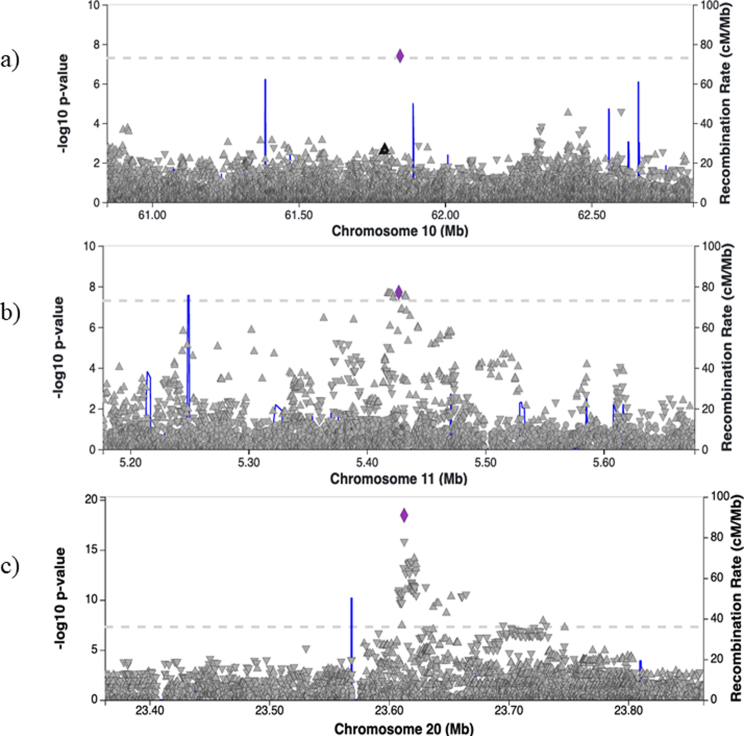
Regional plots for lead SNPs: a) location of rs59288815 in relation to other SNPS on chromosome 10, b) location of rs4277141 in relation to other SNPS on chromosome 11, c) location of rs911119 in relation to other SNPS on chromosome 20. Purple line represents the. lead SNP.

We identified 64 SNPs in our eGFRcys GWAS that were genome-wide significant (P < 5 × 10^−8^ (based on LRT)) and obtained three lead SNPs from these, that is, rs59288815 (*ANK3)*, rs4277141 (*OR51B5*), and rs911119 (*CST3*) (Fig. 2, Table 2 and Fig. S1). All three SNPs are intronic variants in their respective genes. Specifically, rs59288815 is monomorphic in both Europeans and East Asians, whereas rs4277141 and rs911119 are polymorphic in both Europeans and East Asians (see Table 2).

**Table 2:** Lead SNPs from 64 genome-wide significant (P-value <5 × 10-8) SNPs.

Lead SNP	Chr	BP (b37)	Gene	Consequences	EA	NEA	Beta	SE	EAF	P-value	AF-EUR	AF-EAS
rs59288815	10	61,846,677	*ANK3*	Intronic variant	C	T	3.06e-01	5.55e-02	0.053	3.88e-08	0.00	0.00
rs4277141	11	5,426,910	*OR51B5*	Intronic variant	A	G	−1.32e-01	2.340e-02	0.21	1.93e-08	0.49	0.149
rs911119	20	23,612,737	*CST3*	Intronic variant	C	T	−1.74e-01	1.94e-02	0.616	3.51e-19	0.23	0.12

chr: chromosome, BP: base position, EA: effect allele, NEA: non effect allele, SE: standard error, EAF: effect allele frequency, MAF: minor allele frequency, AF_EUR: allele frequency in Europeans, AF-EA: allele frequency in East Asians.

### Replication of the lead single nucleotide polymorphisms

In our study, we identified the rs911119 SNP (P-value = 3.51e-19 (based on LRT)) at the *CST3* locus to having previously been shown to be associated (P-value = 2.10e-202 (based on LRT)) with eGFRcys, with the same direction of effect in European populations by Pattaro et al., 2016 [5] (Table 3). Second, we found rs4277141 SNP at the *OR51B5* locus to be associated (P-value = 1.93e-08) with eGFRcys, which did not replicate (P-value = 0.558 (based on LRT)) in the previous study by Stanzick et al., 2021 in the European population [6]. Finally, the rs59288815 SNP at the *ANK3* locus was also found to be associated with eGFRcys (Table 3), but no prior studies have published our lead SNP to be associated with eGFRcys.

**Table 3:** Replication of lead SNPs.

Gene	Lead SNP	Chr	BP (b37)	EA	NEA	Uganda				Replication			
						Beta	SE	EAF	P-value	Beta	SE	EAF	P-value
*OR51B5*	rs4277141	11	5,426,910	A	G	−1.32e-01	2.340e-02	0.21	1.93e-08	3e-04	5e-04	50.44e-2	0.5588^a^
*CST3*	rs911119	20	23,612,737	C	T	−1.74e-01	1.94e-02	0.616	3.51e-19	−0.07	0.23e-02	7.24e-01	2.10e-202^b^

a Represents the P-value from Stanzick et al., 2021^17^.

b Represents the P-value from Pattaro et al., 2015^16^.

### Comparison of lead SNPs obtained using GFRcrea and eGFRcys GWAS

**Fig. 3:**
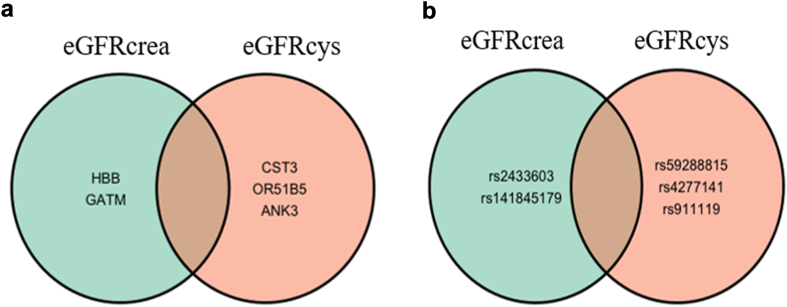
Venn diagrams comparing kidney function results of eGFRcrea and eGFRcys from the GPC. a) Genes associated with kidney function; b) SNPs associated with kidney function.

Upon comparing the SNPs previously identified in the eGFRcrea GWAS [8] with those we identified in the eGFRcys GWAS in the same cohort (UGR), we observed no overlap between the findings from both studies (Fig.3a and b) and no association of our lead SNPs with eGFRcrea (Table 4). We also compared our lead SNPs with global GWAS studies on creatinine and found no overlaps between the previously identified SNPs and our lead SNPs.

**Table 4:** Comparison of lead SNPs obtained from the UGR GWAS eGFRcys with UGR GWAS eGFRcrea.

Gene	Lead SNP	Chr	Bp(b37)	EA	NEA	eGFRcys (Uganda)				eGFRcrea (Uganda)			
						Beta	SE	EAF	P-value	Beta	SE	EAF	P-value
OR51B5	rs4277141	11	5,426,910	A	G	−1.32e-01	2.340e-02	0.21	1.93e-08	−5.71e-02	3.15e-02	0.208	6.97e-02
CST3	rs911119	20	23,612,737	C	T	−1.74e-01	1.94e-02	0.616	3.51e-19	−3.95e-04	2.63e-02	0.608	9.87e-01
ANK3	rs59288815	10	61,846,677	C	T	3.06e-01	5.55e-02	0.053	3.88e-08	6.33e-03	7.16e-02	0.056	9.28e-01

### Fine-mapping of loci attaining genome-wide significance

At the *ANK3* and *CST3* loci, the 99% credible set consisted of only one variant with a 99% posterior probability for each of the two lead SNPs. At the *OR51B5* locus, the 99% credible set consisted of 34 variants, with rs4277141 accounting for 12% of the posterior probability (Table 5).

**Table 5:** Bayesian fine mapping results for the lead SNPs.

rsID	chr/pos (b37)	Closest gene	EA	NEA	af	beta	se	P value	99% Credible sets size	Posterior prob
rs59288815	10:61,846,677	*ANK3*	C	T	0.053	−3.054631e-01	5.550444e-02	3.88e-08	1	0.992
rs4277141	11:5,426,910	*OR51B5*	A	G	0.21	1.319648e-01	2.343220e-02	1.935e-08	34	0.1223
rs911119	20:23,612,737	*CST3*	C	T	0.616	1.741816e-01	1.937286e-02	3.51e-19	1	0.997

chr: chromosome, BP: base position, EA: effect allele, NEA: non effect allele, SE: standard error, af: allele frequency.

### FUMA results

#### Enrichment of input genes in gene sets

**Fig. S2:**
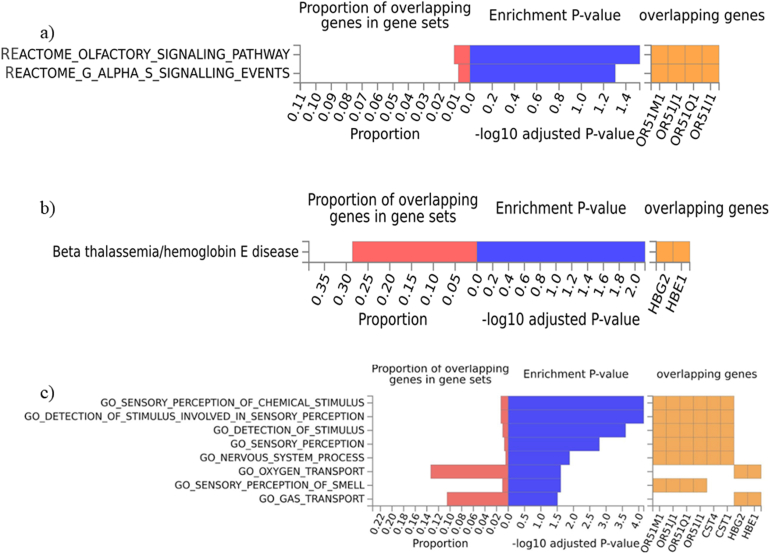
Enrichment of input genes in Gene Sets in FUMA. The -log10 adjusted P-value represents the statistical significance of enrichment after correcting for multiple testing using the Benjamini-Hochberg (BH) method. BH method controls for the false discovery rate, that is to say, the chance of obtaining a false positive due to testing multiple gene sets for enrichment.

From the examination of the enrichment of input genes within gene sets, we identified their correlation with several pathways. These pathways include activities such as cysteine-type endopeptidase inhibitor activity, olfactory signalling pathways, G-alpha-S-signalling events, beta thalassemia/haemoglobin E disease, sensory perception of smell, and oxygen transport (Fig. S2 (a, b, and c), Table S1).

#### Tissue expression and expression quantitative trait loci (eQTL)

**Fig. S3:**
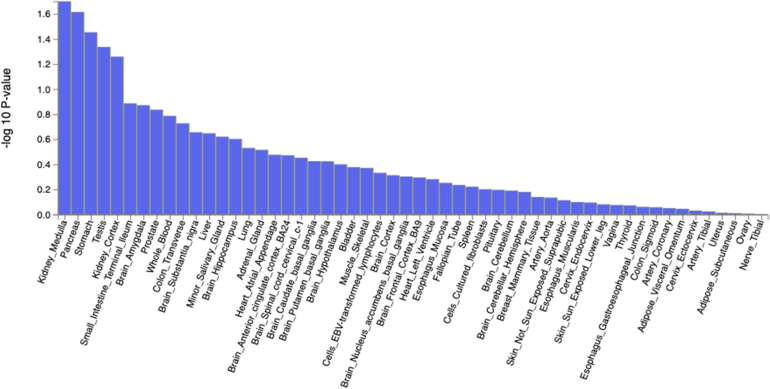
MAGMA Tissue Expression Analysis for GTEx v8 53 tissue types.

Although MAGMA Tissue Expression Analysis on GTEx v8 for the 53 tissue types showed a high tissue expression in the kidney medulla and cortex, the association was not statistically significant (P_bon_ >0.05/53) (Fig. S3).

Furthermore, eGFR association signals do not colocalize with expression quantitative trait loci (eQTLs) in kidney tissue.

#### Gene expression analysis by previous studies

**Fig. S4:**
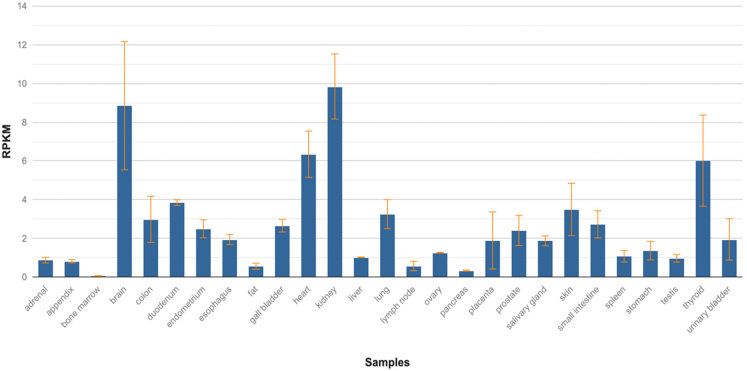
Search results for a study using RNA-seq for 27 tissue samples that showed high A*NK3* gene expression in kidney tissue.^20^

Our literature search identified a study that used RNA-seq on 27 different tissue samples from 95 human individuals in order to determine the tissue-specificity of all protein-coding genes.^20^ The study showed that the *ANK3* gene was highly expressed in the kidney (RPKM (reads per kilobase of transcript per million reads mapped) = 9.846 ± 1.682) and had the highest expression profile in all tissues tested (including the brain) (Fig. S4).

#### Functional mapping and annotation

##### PheWAS

**Fig. S5:**
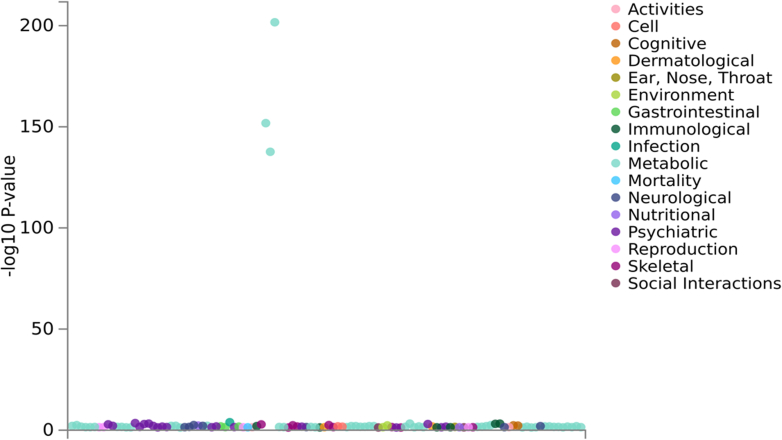
PheWAS results for the rs911119 at the *CST3* locus. **Footnote(s):** (1) Derived from Supplementary Table S2 results. (2) Number of GWASs considered: 4756 (including GWASs in which the searched SNP or gene was not tested, Bonferroni corrected P-value: 1.05e-5). (3) Number of data points in the plot: 114 (Bonferroni corrected P-value: 4.39e-4).

**Fig. S6:**
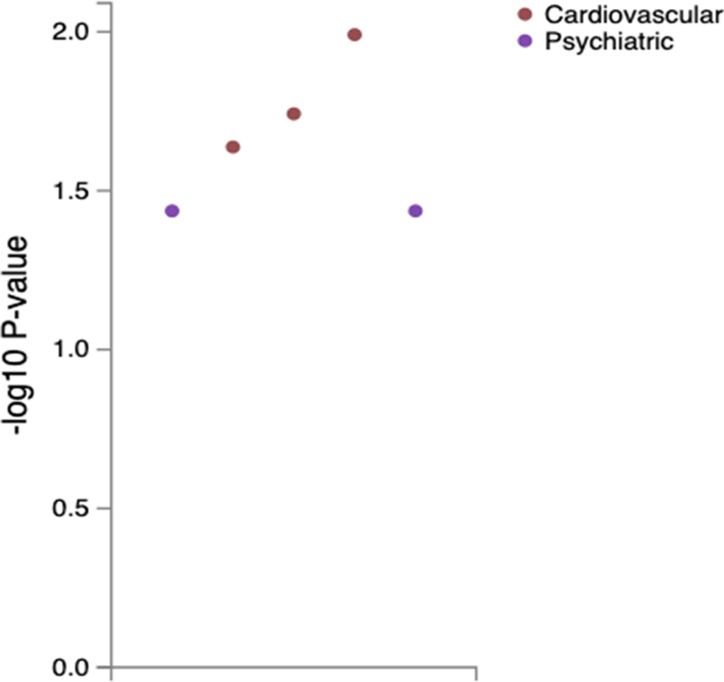
PheWAS results for the rs59288815 at the ANK3 locus. **Footnote(s):** (1) Derived from Supplementary Table S3 results. (2) Number of GWASs considered: 4756 (including GWASs in which the searched SNP or gene was not tested, Bonferroni corrected P-value: 1.05e-5). (3) Number of data points in the plot: 5 (Bonferroni corrected P-value: 1.00e-2).

**Fig. S7:**
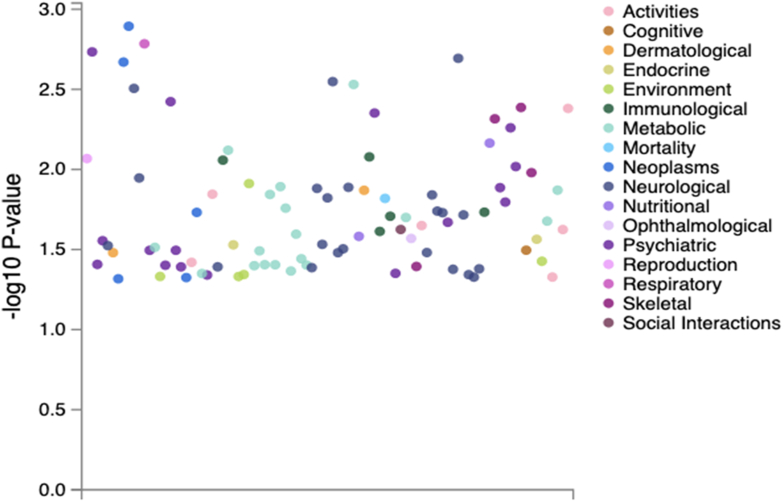
PheWAS results for the rs4277141 SNP at the OR1B5 LOCUS. **Footnote(s):** (1) Derived from Supplementary Table S4 results. (2) Number of GWASs considered: 4756 (including GWASs in which the searched SNP or gene was not tested, Bonferroni corrected P-value: 1.05e-5). (3) Number of data points in the plot: 93 (Bonferroni corrected P-value: 5.38e-4).

The results from GWASATLAS' PheWAS indicate that the *CST3* (rs911119) locus is significantly involved in the metabolism of Cystatin-C (P-value <0.05/4756 (number of GWASs), based logistic regression) (Fig. S5 and Table S2). For the rs59288815 SNP at the *ANK3* locus (Fig. S6 and Table S3) and the *OR51B5* (rs4277141) locus (Fig. S7 and Table S4), there was no significant (P-value <0.05/number of GWASs) association with any trait.

## Discussion

Using data from 5877 UGR participants, we conducted this GWAS to gain an understanding of the underlying genetic risk factors and pathophysiologic mechanisms of kidney disease by exploring beyond traditional biomarkers such as Scr [19]. We identify rs59288815 (*ANK3)* and rs4277141 (*OR51B5*) as loci for kidney function. We also replicate rs911119 (*CST3*) that was previously identified in European ancestry populations. Our findings highlight that using alternative biomarkers such as Cys-C in GWAS of kidney function can enable the discovery of new genetic underpinnings of serum cystatin C-based GFR, a more accurate tool for detecting individuals with low kidney function in Sub-Saharan Africa based on a recent gold-standard validation study of kidney function.^4^

By identifying previously-unreported eGFRcys associations in the *ANK3* (rs59288815) and *OR51B5* (rs4277141) genes, we have uncovered genetic factors contributing to modulation in kidney function among African individuals. Our research also sheds light on pathways of these genetic variants that are linked to eGFRcys. These findings have significant implications for future genetic studies aimed at predicting the risk of kidney diseases among Africans, developing new prevention strategies, detecting, and treating kidney impairments.

First, we found an association with rs59288815, which is an intronic variant in the *ANK3* gene. *ANK3* has been shown to regulate *KCNA1* channel activity in the function of dietary Mg (2+) levels hence regulating renal Mg (2+) reabsorption.^24^ Consistent with our findings, previous studies have shown the *ANK3* gene to be associated with the kidney.^25^ In 2014, Fagerberg et al.*,* used quantitative transcriptomics analysis (RNA-Seq) to classify the tissue-specific expression of genes across major human organs and tissues, and found the *ANK3* gene to be highly expressed in kidney tissue.^20^ Currently, the association of the *ANK3* gene and kidney diseases in humans is not well documented, however the A*NK3* gene is associated with polycystic kidney disease in mice.^26^

Second, rs4277141 is an intronic variant in the *OR51B5* gene that encodes olfactory receptor 51B5. In our pathway analysis using GENE2FUNC, we found *OR51B5* locus to be associated with olfactory signalling. Olfactory receptors (ORs) are mainly odour-sensors in the olfactory epithelium although they are also expressed in several non-sensory tissues.^27^ The olfactory receptors in the kidney have been shown to be involved in blood pressure control and glucose excretion.^28^ Previous studies showed that they also play an essential role in renin secretion, regulation of glomerular filtration, and tubular reabsorption processes.^29^ Despite their role in the normal physiology of the kidney, little is known about their potential effect on renal disorders.^30^ The little we know is that ORs significantly change during the progression of kidney fibrosis.^31^

Third, rs911119 is an intronic variant in the CST3 gene located on chromosome 20. It is the gene which encodes cystatin C which is the biomarker used to derive eGFRcys. Thus, associations between cystatin C and polymorphisms in the CST3 locus may reflect cystatin C production, rather than necessarily kidney function. The *CST3* protein is a widely available extracellular inhibitor of cysteine proteases, found in significant quantities in biological fluids and is present in nearly all body organs.^32^ Its main function is to regulate protease activity, helping to maintain protein balance and preventing excessive protein degradation in different organs. Previous studies have also shown its expression in kidney tissue.^33^ Studies carried out in mice have shown *CST3* to have anti-fibrotic activities by inducing apoptotic cell death and reduced collagen production.^34^

Similar to previous findings in European-ancestry populations,^17^ we also found that the rs911119 SNP at the *CST3* locus is significantly associated with Cys-C concentration in Africans.

In the early GWAS paper from Anna Koettgen,^35^ both cystatin C and creatinine were used, and only those loci which were associated with both were assumed to be kidney function markers.

Though our study size is moderate, we show the same direction of effect for all lead SNPs identified in eGFRcys and in eGFR serum creatinine (Table 4). We think with larger studies' power GWAS, we may be able to report loci which were associated with both eGFRcys and eGFRcrea. Unfortunately, eGFRcrea's performance in Sub-Saharan Africa is so poor that the genetic studies need to be very large indeed to ensure that associations are with kidney function impairment.

No significant eQTLs were detected for any of our lead SNPs by examining several resources for kidney expression, such as GTEx, NephQTL, the Human Kidney eQTL Atlas, and RegulomeDB. This lack of identification may be attributed to the fact that the aforementioned eQTL resources are mainly dominated by European populations. However, the enrichment of input genes in gene-set was associated with G-alpha-S-signalling-events. Interestingly, the G-protein signalling system is biologically relevant to kidney function as it enables the kidney to readily adapt to an ever-changing environment.^36^

The strength of our study is that we have addressed the limitations of the previous GWAS of kidney function in Africa^3^ which used a less reliable biomarker (Scr) and a smaller population size (3288) [3, 4, 8]. We used an alternative and more reliable biomarker (Cys-C) plus a larger sample size (5875) and discovered two plausible independent loci (rs59288815 (*ANK3*) and rs4277141 (*OR51B5*), and replicated findings from other studies (rs911119 (*CST3*)). We also found biologically plausible secondary analysis findings using in silico pathway analysis, tissue and gene expression analysis plus PheWAS.

The limitation of our study is that we analysed samples from one region in Africa, south-western Uganda, which might not be generalisable. For example, the missing replication of the findings from the *ANK3* locus was either because it had a monomorphic or very low allele frequency of the effect allele of the lead SNP in the non-Ugandans, which could be a reason why the lead SNP was not available in the previously published GWAS datasets for replication. We recommend that future studies include a more diverse African population to better represent the African continent.

Another notable limitation of our study is the lack of other existing genotyped biorepositories on kidney function in Africa that use eGFRcys as a biomarker. The absence of similar investigations in Africa limits the comparability and generalizability of our findings to other populations. To address this limitation, we attempted to partially overcome the gap by performing a replication analysis in the same population, utilizing serum creatinine. Although serum creatinine is a commonly used measure for kidney function, it is important to acknowledge that it may not capture the same variants as eGFRcys, and creatinine levels are also driven by any aspect of the creatinine/creatinine production pathway. Thus, while our replication study using serum creatinine adds valuable insights, it is crucial to recognise the potential differences and limitations associated with using an alternative biomarker. The absence of previous investigations utilizing eGFRcys in Africa underscores the need for future research endeavours to further elucidate the genetic underpinnings of kidney function in this region, specifically utilizing the same biomarker for a more comprehensive understanding.

There is also a great need for multi-omics resources in Africa to follow up on significant findings and gain biological insights. Future studies on functional experiments using model organisms in Africa are also necessary to ensure the validation of potentially causal variants.

## Contributors

SF conceptualised the study. RM and TM conducted the main analyses, while SF, OS, ABK, CK and TC accessed and verified the data through rigorous review, reanalysis, and cross-referencing of results. RM wrote the first draft of the manuscript. SF and TC supervised the project. RM, TM OS, ABK, CK, AK, RK, OJS, DJ, JF, CRC, NF, DN, MY, EZ, APM, TC, SF. were involved in discussions of study design and provided expert consult for relevant areas. AK, RK, OJS, DJ, JF, CRC, NF, DN, MY, EZ, APM reviewed the manuscript critically for important intellectual content and conducted editing and rewriting. All authors read and approved the final version of the manuscript.

## Data sharing statement

The data that support the findings of this study are available from the corresponding author upon reasonable request.

## Declaration of interests

None declared.
