# Supplementary material for: Genome-wide association analysis of cystatin-C kidney function in continental Africa
Source: eBioMedicine. 2023 Aug 26;95:104775. doi: 10.1016/j.ebiom.2023.104775 (PMC10474146; doi:10.1016/j.ebiom.2023.104775)
Supplement: Table S1 [file mmc1.docx]

**Table S1**. Total number of enrichment of the input genes

| **Category** | **GeneSet** | **N_genes** | **N_overlap** | **p** | **adjP** | **genes** | **link** |
| --- | --- | --- | --- | --- | --- | --- | --- |
| GO_bp | GO_SENSORY_PERCEPTION_OF_CHEMICAL_STIMULUS | 476 | 6 | 1.81E-08 | 7.07E-05 | OR51M1:OR51J1:OR51Q1:OR51I1:CST4:CST1 | http://www.gsea-msigdb.org/gsea/msigdb/cards/GO_SENSORY_PERCEPTION_OF_CHEMICAL_STIMULUS |
| GO_bp | GO_DETECTION_OF_STIMULUS_INVOLVED_IN_SENSORY_PERCEPTION | 481 | 6 | 1.92E-08 | 7.07E-05 | OR51M1:OR51J1:OR51Q1:OR51I1:CST4:CST1 | http://www.gsea-msigdb.org/gsea/msigdb/cards/GO_DETECTION_OF_STIMULUS_INVOLVED_IN_SENSORY_PERCEPTION |
| GO_bp | GO_DETECTION_OF_STIMULUS | 638 | 6 | 1.03E-07 | 0.000253018 | OR51M1:OR51J1:OR51Q1:OR51I1:CST4:CST1 | http://www.gsea-msigdb.org/gsea/msigdb/cards/GO_DETECTION_OF_STIMULUS |
| GO_bp | GO_SENSORY_PERCEPTION | 916 | 6 | 8.77E-07 | 0.001610635 | OR51M1:OR51J1:OR51Q1:OR51I1:CST4:CST1 | http://www.gsea-msigdb.org/gsea/msigdb/cards/GO_SENSORY_PERCEPTION |
| GO_bp | GO_NERVOUS_SYSTEM_PROCESS | 1362 | 6 | 8.94E-06 | 0.013144875 | OR51M1:OR51J1:OR51Q1:OR51I1:CST4:CST1 | http://www.gsea-msigdb.org/gsea/msigdb/cards/GO_NERVOUS_SYSTEM_PROCESS |
| GO_bp | GO_OXYGEN_TRANSPORT | 15 | 2 | 2.06E-05 | 0.024482811 | HBG2:HBE1 | http://www.gsea-msigdb.org/gsea/msigdb/cards/GO_OXYGEN_TRANSPORT |
| GO_bp | GO_SENSORY_PERCEPTION_OF_SMELL | 407 | 4 | 2.33E-05 | 0.024482811 | OR51M1:OR51J1:OR51Q1:OR51I1 | http://www.gsea-msigdb.org/gsea/msigdb/cards/GO_SENSORY_PERCEPTION_OF_SMELL |
| GO_bp | GO_GAS_TRANSPORT | 19 | 2 | 3.35E-05 | 0.030744935 | HBG2:HBE1 | http://www.gsea-msigdb.org/gsea/msigdb/cards/GO_GAS_TRANSPORT |
| GWAScatalog | Beta thalassemia/hemoglobin E disease | 7 | 2 | 4.12E-06 | 0.007480781 | HBG2:HBE1 |  |
| GO_cc | GO_HAPTOGLOBIN_HEMOGLOBIN_COMPLEX | 11 | 2 | 1.08E-05 | 0.006475444 | HBG2:HBE1 | http://www.gsea-msigdb.org/gsea/msigdb/cards/GO_HAPTOGLOBIN_HEMOGLOBIN_COMPLEX |
| GO_cc | GO_HEMOGLOBIN_COMPLEX | 12 | 2 | 1.29E-05 | 0.006475444 | HBG2:HBE1 | http://www.gsea-msigdb.org/gsea/msigdb/cards/GO_HEMOGLOBIN_COMPLEX |
| Cancer_modules | MODULE_164 | 60 | 3 | 2.43E-06 | 0.001045848 | CST3:CST4:CST1 | http://www.gsea-msigdb.org/gsea/msigdb/cards/MODULE_164 |
| Positional_gene_sets | chr11p15 | 297 | 6 | 1.07E-09 | 3.20E-07 | HBG2:HBE1:OR51M1:OR51J1:OR51Q1:OR51I1 | http://www.gsea-msigdb.org/gsea/msigdb/cards/chr11p15 |
| Positional_gene_sets | chr20p11 | 55 | 3 | 1.86E-06 | 0.000278449 | CST3:CST4:CST1 | http://www.gsea-msigdb.org/gsea/msigdb/cards/chr20p11 |
| GO_mf | GO_CYSTEINE_TYPE_ENDOPEPTIDASE_INHIBITOR_ACTIVITY | 57 | 3 | 2.08E-06 | 0.003415532 | CST3:CST4:CST1 | http://www.gsea-msigdb.org/gsea/msigdb/cards/GO_CYSTEINE_TYPE_ENDOPEPTIDASE_INHIBITOR_ACTIVITY |
| GO_mf | GO_HAPTOGLOBIN_BINDING | 10 | 2 | 8.83E-06 | 0.007259083 | HBG2:HBE1 | http://www.gsea-msigdb.org/gsea/msigdb/cards/GO_HAPTOGLOBIN_BINDING |
| GO_mf | GO_OLFACTORY_RECEPTOR_ACTIVITY | 380 | 4 | 1.78E-05 | 0.007332583 | OR51M1:OR51J1:OR51Q1:OR51I1 | http://www.gsea-msigdb.org/gsea/msigdb/cards/GO_OLFACTORY_RECEPTOR_ACTIVITY |
| GO_mf | GO_OXYGEN_CARRIER_ACTIVITY | 14 | 2 | 1.78E-05 | 0.007332583 | HBG2:HBE1 | http://www.gsea-msigdb.org/gsea/msigdb/cards/GO_OXYGEN_CARRIER_ACTIVITY |
| GO_mf | GO_ENDOPEPTIDASE_REGULATOR_ACTIVITY | 174 | 3 | 5.96E-05 | 0.019597611 | CST3:CST4:CST1 | http://www.gsea-msigdb.org/gsea/msigdb/cards/GO_ENDOPEPTIDASE_REGULATOR_ACTIVITY |
| GO_mf | GO_PEPTIDASE_REGULATOR_ACTIVITY | 209 | 3 | 0.000102677 | 0.028150505 | CST3:CST4:CST1 | http://www.gsea-msigdb.org/gsea/msigdb/cards/GO_PEPTIDASE_REGULATOR_ACTIVITY |
| GO_mf | GO_OXYGEN_BINDING | 36 | 2 | 0.000122778 | 0.02885279 | HBG2:HBE1 | http://www.gsea-msigdb.org/gsea/msigdb/cards/GO_OXYGEN_BINDING |
| GO_mf | GO_MOLECULAR_CARRIER_ACTIVITY | 41 | 2 | 0.000159611 | 0.032820069 | HBG2:HBE1 | http://www.gsea-msigdb.org/gsea/msigdb/cards/GO_MOLECULAR_CARRIER_ACTIVITY |
| Computational_gene_sets | MODULE_164 | 60 | 3 | 2.43E-06 | 0.00208199 | CST3:CST4:CST1 | http://www.gsea-msigdb.org/gsea/msigdb/cards/MODULE_164 |
| Reactome | REACTOME_OLFACTORY_SIGNALING_PATHWAY | 391 | 4 | 1.99E-05 | 0.029854658 | OR51M1:OR51J1:OR51Q1:OR51I1 | http://www.gsea-msigdb.org/gsea/msigdb/cards/REACTOME_OLFACTORY_SIGNALING_PATHWAY |
| Reactome | REACTOME_G_ALPHA_S_SIGNALLING_EVENTS | 531 | 4 | 6.60E-05 | 0.049488688 | OR51M1:OR51J1:OR51Q1:OR51I1 | http://www.gsea-msigdb.org/gsea/msigdb/cards/REACTOME_G_ALPHA_S_SIGNALLING_EVENTS |
| Canonical_Pathways | REACTOME_OLFACTORY_SIGNALING_PATHWAY | 391 | 4 | 1.99E-05 | 0.043796126 | OR51M1:OR51J1:OR51Q1:OR51I1 | http://www.gsea-msigdb.org/gsea/msigdb/cards/REACTOME_OLFACTORY_SIGNALING_PATHWAY |
