# Supplementary material for: Genome-wide association analysis of cystatin-C kidney function in continental Africa
Source: eBioMedicine. 2023 Aug 26;95:104775. doi: 10.1016/j.ebiom.2023.104775 (PMC10474146; doi:10.1016/j.ebiom.2023.104775)
Supplement: Table S2 [file mmc2.docx]

**Table S2**: PheWAS results for the rs911119 at the *CST3* locus

| **atlas ID** | **PMID** | **Year** | **Domain** | **Trait** | **P-value** | **N** | **EA** | **NEA** |
| --- | --- | --- | --- | --- | --- | --- | --- | --- |
| 194 | 26831199 | 2016 | Metabolic | Estimated glomerular filtration rate based on cystain C | 2.10E-202 | 33152 | C | T |
| 4064 | 28452372 | 2017 | Metabolic | Estimated glomerular filtration rate based on cystain C | 1.57E-152 | 24061 | C | T |
| 202 | 20383146 | 2010 | Metabolic | Estimated glomerular filtration rate based on cystain C | 2.30E-138 | 20957 | C | T |
| 3684 | 31427789 | 2019 | Infection | Diagnoses - main ICD10: R31 Hematuria | 0.0001133 | 300791 | T | C |
| 3759 | 31427789 | 2019 | Psychiatric | Anxiety - Reccent feelings or nervousness or anxiety | 0.0003023 | 126325 | T | C |
| 3768 | 31427789 | 2019 | Psychiatric | Anxiety - Recent trouble relaxing | 0.0005577 | 126422 | T | C |
| 510 | 24816252 | 2014 | Metabolic | Lipid::Inositol metabolism::chiro-inositol | 0.0005581 | 2729 | T | C |
| 3865 | 27863252 | 2016 | Immunological | Red cell distribution width (two-way meta) | 0.00058252 | 131520 | C | T |
| 3901 | 27863252 | 2016 | Immunological | Red cell distribution width (three-way meta) | 0.0007333 | 171529 | C | T |
| 4013 | 29662059 | 2018 | Psychiatric | Major depressive disorder (ICD-coded) | 0.0009074 | 217584 | C | T |
| 3762 | 31427789 | 2019 | Psychiatric | Anxiety - Recent inability to stop or control worying | 0.000923 | 126300 | T | C |
| 3657 | 31427789 | 2019 | Psychiatric | Alcohol - Alcohol drinker status: Previous vs Current | 0.001298 | 373560 | C | T |
| 4328 | 30598549 | 2018 | Skeletal | Estimated bone mineral density from heel ultrasounds | 0.0014 | 426824 | T | C |
| 4718 | 288626 | 2019 | Neurological | Cingulum (hippocampus) mode of anisotropy | 0.003017 | 17706 | T | C |
| 4175 | 30048462 | 2018 | Skeletal | Heel bone mineral density | 0.0031 | 394929 | T | C |
| 1119 | 27918534 | 2017 | Metabolic | Pericardial adipose tissue volume (female) | 0.0032 | 6362 | NA | NA |
| 80 | 22504420 | 2012 | Skeletal | Femoral Neck BMD | 0.003847 | 32961 | C | T |
| 3432 | 31427789 | 2019 | Cognitive | Symbol digit substitution test - Number of symbol digit matches attempted | 0.004104 | 95669 | T | C |
