# Supplementary material for: Genome-wide association analysis of cystatin-C kidney function in continental Africa
Source: eBioMedicine. 2023 Aug 26;95:104775. doi: 10.1016/j.ebiom.2023.104775 (PMC10474146; doi:10.1016/j.ebiom.2023.104775)
Supplement: Table S3 [file mmc3.docx]

**Table S3**: PheWAS results for the rs59288815 at the *ANK3* locus

| **atlas ID** | **PMID** | **Year** | **Domain** | **Trait** | **P-value** | **N** | **EA** | **NEA** |
| --- | --- | --- | --- | --- | --- | --- | --- | --- |
| 4195 | 31217584 | 2019 | Cardiovascular | Diastolic Blood Pressure | 0.01811371 | 35433 | C | T |
| 4196 | 31217584 | 2019 | Cardiovascular | Hypertension | 0.01021202 | 49141 | C | T |
| 4270 | 30867560 | 2019 | Psychiatric | Anxiety/tension factors | 0.03661845 | 270059 | T | C |
| 4271 | 30867560 | 2019 | Psychiatric | Worry/vulnerability factors | 0.03661845 | 270059 | T | C |
| 4361 | 30061737 | 2018 | Cardiovascular | Atrial fibrillation | 0.02304 | 1030836 | C | T |

EA: effect allele; NEA: non-effect allele; N: number of samples
