# Supplementary material for: Genome-wide association analysis of cystatin-C kidney function in continental Africa
Source: eBioMedicine. 2023 Aug 26;95:104775. doi: 10.1016/j.ebiom.2023.104775 (PMC10474146; doi:10.1016/j.ebiom.2023.104775)
Supplement: Table S4 [file mmc4.docx]

**Table S4**: PheWAS results for the rs4277141 SNP at the *OR51B5* locus

| **Atlas ID** | **PMID** | **Year** | **Domain** | **Trait** | **P-value** | **N** | **EA** | **NEA** |
| --- | --- | --- | --- | --- | --- | --- | --- | --- |
| 3659 | 31427789 | 2019 | Neoplasms | Cancer register - Type of cancer: ICD10: C50 Malignant neoplasm of breast | 0.001277 | 54778 | G | A |
| 3371 | 31427789 | 2019 | Respiratory | Chest pain or discomfort when walking uphill or hurrying | 0.001643 | 48190 | A | G |
| 3728 | 31427789 | 2019 | Psychiatric | Anxiety - Ever worried more than most people would in similar situation | 0.001846 | 106215 | A | G |
| 4592 | 31676860 | 2019 | Neurological | Right pars opercularis | 0.002025 | 21821 | G | A |
| 3663 | 31427789 | 2019 | Neoplasms | Cancer register - Behaviour of cancer tumour: Malignant, primary site | 0.002138 | 61413 | G | A |
| 4556 | 31676860 | 2019 | Neurological | Left lingual | 0.002831 | 21821 | G | A |
| 4304 | 30664634 | 2019 | Metabolic | Legs-leg fat ratio (female) | 0.002949 | 195068 | A | G |
| 4509 | 31676860 | 2019 | Neurological | Cerebellar vermal lobules VIII X | 0.003121 | 19629 | A | G |
| 3738 | 31427789 | 2019 | Psychiatric | Depression - Lifetime number of depressed periods | 0.003776 | 57986 | G | A |
| 3187 | 31427789 | 2019 | Skeletal | Standing height | 0.004103 | 385748 | G | A |
| 3337 | 31427789 | 2019 | Activities | Years since last breast cancer screening / mammogram (female) | 0.004161 | 162926 | A | G |
| 3755 | 31427789 | 2019 | Psychiatric | Mental distress - Ever sought or received professional help for mental distress | 0.004447 | 126402 | A | G |
| 3412 | 31427789 | 2019 | Skeletal | Sitting height | 0.004832 | 385393 | G | A |
| 4343 | 30531941 | 2018 | Psychiatric | Sleep sedentary | 0.0055 | 91105 | G | A |
| 3254 | 31427789 | 2019 | Nutritional | Salt added to food | 0.006852 | 386322 | A | G |
| 3186 | 31427789 | 2019 | Metabolic | Hip circumference | 0.007594 | 385887 | G | A |
| 4419 | 30053915 | 2018 | Immunological | Measles seropositivity | 0.00835185 | 1000 | A | G |
| 3347 | 31427789 | 2019 | Reproduction | Age started oral contraceptive pill (female) | 0.008559 | 165121 | A | G |
| 3842 | 27863252 | 2016 | Immunological | Granulocyte percentage of myeloid white cells (two-way meta) | 0.00875409 | 130543 | A | G |
|  | 30531941 | 2018 | Psychiatric | Sleep sedentary (conditioning sex and BMI) | 0.0096 | 91105 | G | A |
